# Symportin 1 chaperones 5S RNP assembly during ribosome biogenesis by occupying an essential rRNA-binding site

**DOI:** 10.1038/ncomms7510

**Published:** 2015-04-07

**Authors:** Fabiola R. Calviño, Satyavati Kharde, Alessandro Ori, Astrid Hendricks, Klemens Wild, Dieter Kressler, Gert Bange, Ed Hurt, Martin Beck, Irmgard Sinning

**Affiliations:** 1 Heidelberg University Biochemistry Center, Im Neuenheimer Feld 328, D-69120 Heidelberg, Germany; 2 European Molecular Biology Laboratory, Structural and Computational Biology Unit, Meyerhofstrasse 1, 69117 Heidelberg, Germany; 3 Unit of Biochemistry, Department of Biology, University of Fribourg, Chemin du Musée 10, CH-1700 Fribourg, Switzerland

## Abstract

During 60S biogenesis, mature 5S RNP consisting of 5S RNA, RpL5 and RpL11, assembles into a pre-60S particle, where docking relies on RpL11 interacting with helix 84 (H84) of the 25S RNA. How 5S RNP is assembled for recruitment into the pre-60S is not known. Here we report the crystal structure of a ternary symportin Syo1–RpL5-N–RpL11 complex and provide biochemical and structural insights into 5S RNP assembly. Syo1 guards the 25S RNA-binding surface on RpL11 and competes with H84 for binding. Pull-down experiments show that H84 releases RpL11 from the ternary complex, but not in the presence of 5S RNA. Crosslinking mass spectrometry visualizes structural rearrangements on incorporation of 5S RNA into the Syo1–RpL5–RpL11 complex supporting the formation of a pre-5S RNP. Our data underline the dual role of Syo1 in ribosomal protein transport and as an assembly platform for 5S RNP.

## Abstract (web summary)

Biogenesis of the 80S ribosome involves more than 200 pre-ribosomal factors, which ensure the sequential assembly of ribosomal proteins and RNAs. Here the authors show that the nuclear transport adaptor Syo1 shields the 5S RNP-docking site on RpL11 before incorporation into the pre-60S through molecular mimicry.

## Graphical abstract

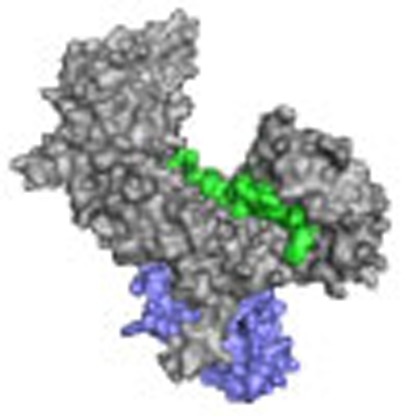

Ribosomes catalyse protein synthesis in the cytoplasm^1^, but eukaryotic ribosome assembly occurs predominantly in the nucleus and involves more than 200 pre-ribosomal factors. Ribosome biogenesis requires the ordered assembly of ∼80 ribosomal proteins (r-proteins) and four ribosomal RNAs (rRNAs)^2^,^3^,^4^. These r-proteins need to be imported from the cytoplasm into the nucleus, where they will sequentially assemble onto pre-ribosomal particles^5^. Many r-proteins form functional clusters within the ribosome^6^ or assemble at distinct entry points during ribosome assembly^7^. Typical examples are the r-proteins RpL5 and RpL11 which, together with the 5S RNA, form the 5S RNP. Nuclear import of RpL5 and RpL11 and their transfer onto the 5S RNA is synchronized by the recently identified symportin Syo1 (ref. 8). It concomitantly binds RpL5 and RpL11 in the cytoplasm, traverses the nuclear pore via the import receptor Kap104 and transfers its cargo onto the 5S RNA. Syo1 forms an elongated α-solenoid by an unusual combination of four complete ARM repeats and six HEAT repeats. Recently, we have shown that Syo1 recognizes RpL5 via a linear motif at the N-terminus, which in the 5S RNP interacts with the 5S RNA, similar to other nuclear transport proteins and their cargos (for example, Nup2 (ref. 9) and importin α (ref. 10); ref. 5). However, how Syo1 achieves concomitant binding of its two cargos and thus may assist 5S RNP assembly remained enigmatic.

To date, only little data are available for the 5S RNP assembly, which seems to involve a number of factors before the complex is docked to the large ribosomal subunit assembly intermediate (pre-60S) in an early stage of 60S maturation^8^,^11^. Recently, a eukaryotic pre-60S was characterized by cryo-electron microscopy, and notably, 5S RNP is rotated by about 180° compared with its position in mature 60S. Its docking relies on RpL11 interacting with helix 84 (H84) of the 25S RNA^12^. How 5S RNP is assembled for recruitment into the pre-60S is still unknown.

Here we report the crystal structure of the Syo1–RpL5–RpL11 complex, which shows that both cargos of Syo1 simultaneously bind to opposing sites of its α-solenoid structure. Syo1 chaperones 5S RNP and masks the major binding site of RpL11 for the 60S ribosomal subunit. Pull-down experiments and crosslinking mass spectrometry (XL-MS) provide insights into the 5S RNP assembly and underline a dual role of Syo1 in r-protein transport and ribosome biogenesis.

## Results

### Structure determination

**Figure 1:**
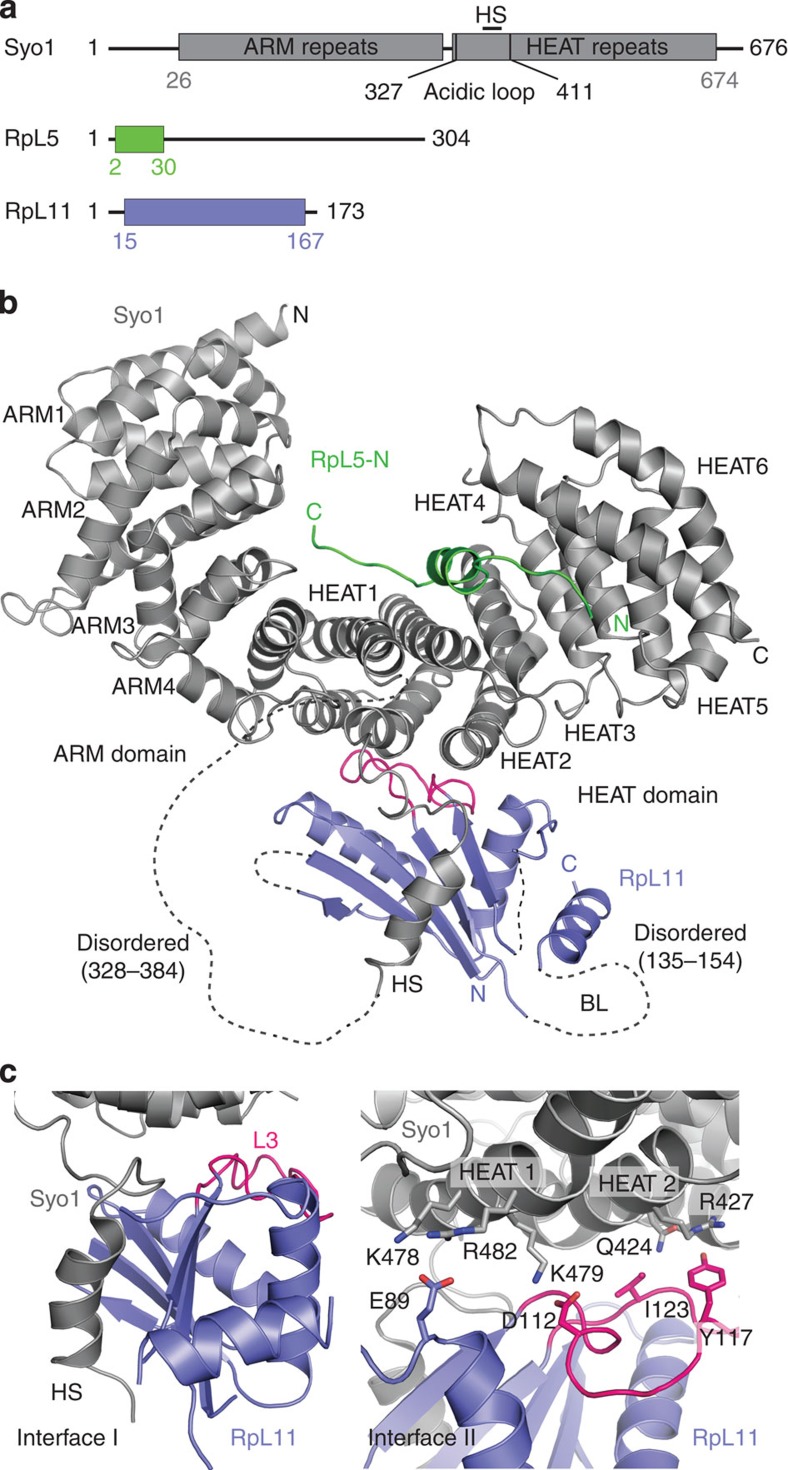
Structure of the Syo1–RpL5-N–RpL11 complex. (**a**) Domain architecture of Syo1 (75 kDa), RpL5 (35 kDa) and RpL11 (25 kDa). Domains present in the crystal structure are given by residue numbers and are highlighted in colour. BL, basic loop; HS, helical segment from the acidic loop. (**b**) Overall structure of Syo1 (grey) in complex with RpL5-N (green) and RpL11 (blue). Disordered regions are shown as dashed lines, with residue numbers. N- and C-termini are indicated. (**c**) Close up of Syo1 (grey) and RpL11 (blue) interactions (interface I and II). Loop L3 is highlighted in red; HEAT repeats 1 and 2 are labelled.

As a first step to understand how RpL11 interacts within the Syo1–RpL5–RpL11 complex, we determined the crystal structure of *Chaetomium thermophilum* RpL11 (residues 15–167) in complex with RpL5-N (residues 2–30) and Syo1 (residues 26–674; Fig. 1a,b and Table 1). Proteins from this thermophilic fungus often show improved biochemical properties, and consistent mutational paths predict eukaryotic thermostability^13^,^14^. The Syo1–RpL5-N–RpL11 complex was produced in *Escherichia coli* and purified by affinity chromatography followed by size-exclusion chromatography (SEC). Crystallization was performed at 291 K by the sitting-drop vapour-diffusion method. Crystals belong to the orthorhombic space group P2_1_2_1_2_1_ with unit cell parameters of *a*=60.1, *b*=106.0 and *c*=147.2 Å (Table 1). One Syo1–RpL5-N–RpL11 complex is present in the crystal asymmetric unit, corresponding to a Matthews coefficient^15^ of 2.1 Å^2^ per Da and a solvent content of ≈41%. The structure was solved by molecular replacement using the coordinates of Syo1 (ref. 8) and of RpL11 from the yeast ribosome^16^, and refined to a resolution of 3.4 Å with *R*_work_/*R*_free_ values of 24.0 and 29.9%, respectively (Table 1). The structure is well defined for Syo1 and RpL5-N, while RpL11 appears more flexible probably due to missing crystal contacts. A large part of the acidic loop in Syo1 (residues 328–384) and the basic loop in RpL11 (residues 135–154) are disordered.

### Crystal structure of the Syo1–RpL5-N–RpL11 complex

The symportin Syo1 is an α-solenoid that binds RpL5 and RpL11 on opposite sites of the HEAT repeats, whereas the ARM repeats are not involved in cargo binding (Fig. 1b). RpL5-N (residues 2–30) interacts as a linear motif with an elongated structure and a short helix (Tyr12 to Phe16). In the ternary complex, residues Phe3 to Asn9 are relocated from HEAT repeat 1 and 2 to HEAT repeat 3 and 4 compared with the previous structure^8^ (Supplementary Fig. 1a), and RpL5-N becomes more solvent exposed. Although relocation might reflect the different crystallographic environments, it might facilitate release of RpL5 from Syo1 for 5S RNA binding and further 5S RNP assembly. RpL11 localizes at the convex side of Syo1 with about 1,160 Å^2^ buried surface area (Fig. 1b,c). RpL11 in the trimeric complex is basically identical to its structure as part of the 80S ribosome indicating that the globular part of RpL11 binds as a rigid body (Supplementary Fig. 1b). The Syo1–RpL11 interface comprises two main parts. The first one (interface I) involves a helical segment in the acidic loop of Syo1 (HS, amino acids Glu389 to Gly399) and the carboxyl C-terminal end of the acidic loop, which was disordered in the Syo1–RpL5-N structure (Fig. 1c, left). The RpL11 β-sheet forms a groove, which accommodates the Syo1-HS (Fig. 1b,c). Interface I is crucial for RpL11 binding as indicated by deletion variants and HX-MS analysis^8^. The second one (interface II) involves the Syo1 HEAT repeats 1 to 3 (Fig. 1c, right). Mutational analysis shows that RpL11 loop L3 (in particular, Asp112, Tyr117 and Ile123) and Glu89 play important roles in this interface (Fig. 1c; Supplementary Figs 2 and 3). However, when interface I is intact, the Syo1–RpL11 interaction cannot be completely abolished by changes in interface II. Overall, we show how Syo1 binds two r-proteins simultaneously and that the interface I, employing Syo1-HS, is the main determinant for interaction with RpL11.

Syo1 is a dynamic α-solenoid and the binding of RpL5 and RpL11 induces adjustments in the superhelix. Syo1 alone shows a ‘closed' conformation, which turns to an ‘open' conformation on its interaction with RpL5-N^8^ (Supplementary Fig. 4, top panel). Opening corresponds to a rotation of about 13° around a hinge axis almost parallel to the RpL5-N helix inserted between the ARM and the HEAT repeats. Superposition of the Syo1–RpL5-N complex with the ternary Syo1–RpL5-N–RpL11 structure reveals minor further adjustments reflecting that RpL11 binds to the outside of the solenoid (root mean squared deviation of 0.8 Å for 527 Cα-atoms; Supplementary Fig. 4). The relocation of the RpL5 amino N-terminal residues observed in the presence of RpL11 is intriguing and might correlate with the slight closure of Syo1 in the ternary complex (Supplementary Fig. 4, middle panel). Taken together, our data show how the binding of the two cargos affects the Syo1 conformation and underline the intrinsic flexibility known as an important functional feature of α-solenoid structures^17^.

### Syo1-HS occupies the 25S RNA-binding site on RpL11

**Figure 2:**
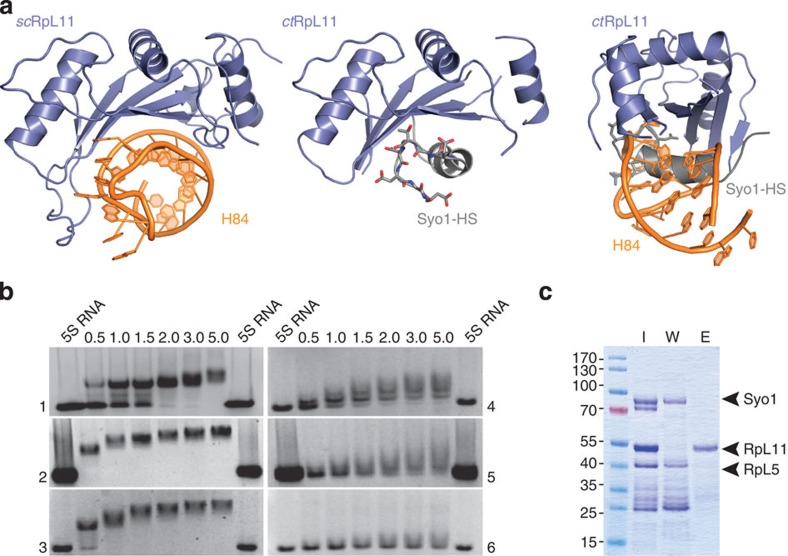
Syo1 mimics 25S RNA (helix 84) binding to RpL11. (**a**) RpL11 interaction with Syo1 (this study; middle), with helix 84 (as part of the ribosome; left^16^) are compared by superposition of both complexes (right; rotated by 90°). Syo1-HS and H84 share the binding site on RpL11 and read out its fold. (**b**) Binding of 5S RNA was tested by EMSAs. The titrations are shown for Syo1–RpL5–RpL11 (panel 1), Syo1–RpL5–RpL11ΔBL (panel 2), Syo1–RpL5ΔC–RpL11ΔBL (panel 3), Syo1–RpL5-N–RpL11 (panel 4), Syo1–RpL11 (panel 5) and Syo1–RpL11ΔBL complexes (panel 6). ‘RNA' lanes are free of protein to provide a base line for the shift. The protein/RNA ratio is given at the top. (**c**) Pull-down analysis of H84 interaction with the Syo1–RpL5–RpL11 complex (all proteins are full length). A GST-RpL11 variant was used to immobilize the complex on a GSTrap HP column (lane I). Washing with H84 releases Syo1–RpL5 (lane W) from GST-RpL11 (lane E).

Docking of the 5S RNP to the 60S ribosomal subunit occurs early in ribosome biogenesis and involves an interaction between RpL11 and helix 84 (H84) of the 25S RNA^12^,^16^. Superposition of our ternary Syo1 complex with 5S RNP bound to the 60S ribosomal subunit^16^ shows that H84 and the Syo1-HS share the same binding site on RpL11 (Fig. 2a, left and middle). More detailed analysis shows that the Syo1-HS not only occupies the H84 binding site, but also shares molecular details with its RNA counterpart. Specifically, superposition reveals a distinct bent in the Syo1-HS, which mirrors the minor groove of H84 and subsequent acidic loop residues 401–407 superimpose with the tip of H84 (Fig. 2a, right; Supplementary Fig. 5). Overall, the shape and charge distribution of the Syo1-HS and H84 are very similar. However, the given resolution of both structures does not allow for a more detailed analysis of ‘molecular mimicry' of their interactions with RpL11. Our results point to a more elaborate function of Syo1 in 5S RNP biogenesis, as it protects the 25S RNA-binding site on RpL11 by offering a structural placeholder for H84. Thereby, Syo1 shields an essential binding site needed for 5S RNP docking to the pre-60S particle early in 60S maturation.

### Analysis of 5S RNA binding by EMSAs

At present, it is not known how 5S RNP assembly proceeds in the nucleolus and how Syo1 is involved. Association of the 5S RNA with the ternary complex does not release Syo1, but generates a tetrameric 5S RNP intermediate (pre-5S RNP) consisting of Syo1–RpL5–RpL11 and the 5S RNA^8^. The 5S RNA-binding activity of the Syo1–RpL5–RpL11 complex was monitored by electrophoretic mobility shift assays (EMSAs; Fig. 2b). RNA was folded according to an established protocol^18^. The assays confirmed that Syo1 does not bind the 5S RNA (Supplementary Fig. 6a), while in the presence of RpL5 and RpL11, a pre-5S RNP is formed (Fig. 2b, panel 1). A number of truncation variants of RpL5 and RpL11 were employed to analyse the individual contribution of their 5S RNA-interaction sites (Fig. 2b, panels 1 to 6). In the mature 5S RNP, the RpL5 N- and C-termini clamp the RNA and RpL11 interacts mainly via its basic loop (comprising residues Arg142 to Lys146). The EMSAs showed that the Syo1–RpL11 complex is not able to recruit the 5S RNA, although the basic loop is solvent exposed in the structure. Efficient binding requires both RpL5 and RpL11, with RpL5 N-terminus and globular domain providing the major binding site (Supplementary Fig. 6c). Overall, these data suggest that in the pre-5S RNP the RNA is clamped by the RpL5 N- and C-termini probably as in the mature 5S RNP.

### Analysis of RpL11 interactions

Since Syo1-HS and H84 utilize the same binding site on RpL11, we first used EMSAs to analyse the interaction of H84 with the ternary complex (Supplementary Fig. 6b). H84 does not bind to Syo1 alone, but interacts with the ternary complex, although less efficient compared with 5S RNA. To test whether H84 competes with Syo1 for RpL11 binding, we used a glutathione *S*-transferase (GST)-tagged RpL11 variant in an *in vitro* pull-down approach (Fig. 2c). On addition of H84, the ternary complex dissociates into Syo1–RpL5 and H84 bound to GST-RpL11 (Fig. 2c, Supplementary Fig. 7). The release of RpL11 by H84 shows that interface I in the ternary complex (Fig. 1c) provides the main contact between Syo1 and RpL11, and that there is no significant interaction between RpL5 and RpL11. Syo1-HS and H84 compete for the same binding site on RpL11, and Syo1 binding to RpL11 might ensure the presence of an intact binding site for H84 to allow the successful recruitment of the 5S RNP into the pre-60S.

To test whether 5S RNP assembly proceeds in the presence of Syo1 (as indicated by the EMSAs), we performed the competition experiment with H84 now in the presence of the 5S RNA. In mature 5S RNP, RpL11 interacts with the 5S RNA as well as with RpL5 (ref. 16). Therefore, H84 should not be able to release RpL11 if these contacts are established already in pre-5S RNP. Indeed, using a Syo1-FLAG variant, we observed that the addition of H84 to the tetrameric complex did not dissociate RpL11 (Supplementary Fig. 8). Instead, H84 is now recruited into the pre-5S RNP. RpL11 probably binds to the 5S RNA by its basic loop and by residues from its globular domain as described^16^,^19^. Overall, our data indicate that 5S RNP assembly proceeds in the presence of Syo1.

### XL-MS experiments

To gain insights into the structural rearrangements underlying the pre-5S RNP assembly, we performed XL-MS experiments on the Syo1–RpL5–RpL11 complex with and without the 5S RNA. The purified complexes were crosslinked with the lysine-specific reagent disuccimidyl suberate and crosslinked peptides were identified by high-resolution tandem mass spectrometry using the xQuest and xProphet software^20^,^21^,^22^. Samples with and without RNA were processed in parallel and analysed separately. In total, we identified 67 intralinks (crosslinks within the same polypeptide) and 35 interlinks (crosslinks between two polypeptides), corresponding to 32 and 23 unique restraints, respectively (Table 2). Selected crosslinks were quantitatively compared between samples by label-free quantification based on manual peak extraction (see Supplementary Fig. 9 and Table 2).

**Figure 3:**
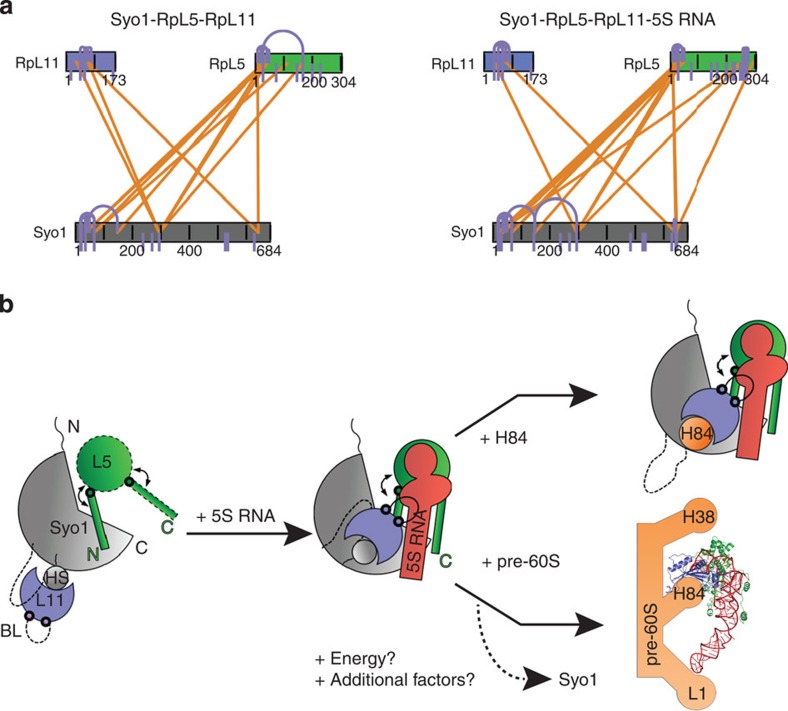
Syo1 serves as a platform for 5S RNP assembly. (**a**) Inter- (orange) and intraprotein (blue lines) crosslinks detected on the Syo1 complex in the absence (left) or presence (right) of the 5S RNA. Quantified crosslinks are shown in Table 2 and Supplementary Fig. 9. (**b**) Model for the role of Syo1 in 5S RNP assembly (left and middle panel). H84 is recruited into the tetrameric pre-5S RNP (right panel, top) by interaction with RpL11 as used in 5S RNP docking to the pre-60S particle (right panel, bottom)^12^. Parts of the proteins absent or disordered in the ternary complex are represented by dotted lines. Hinge points (represented as thick circles) in the proteins allow for rearrangements (indicated by arrows). Syo1 (grey), RpL5 (green), RpL11 (blue), 5S RNA (red) and helix 84 (H84, orange). The model is based on the ternary complex (this study) and the 5S RNP as part of the yeast ribosome (PDB codes: 3U5E, 3U5D)^16^. H84 interacts with the 5S RNP in the pre-60S and in the mature ribosome in the same manner.

The structure of the Syo1–RpL5-N–RpL11 complex (this study) and of the mature 5S RNP from the 80S ribosome^16^ provide constraints for a detailed analysis of the crosslink results. In the absence of RNA, the structure of the ternary complex in solution is consistent with the crystal structure as indicated by the formation of specific crosslinks between RpL5 and RpL11 with Syo1, while crosslinks between RpL5 and RpL11 were not observed (Table 2, Fig. 3a, see Supplementary Fig. 10). A number of crosslinks (Syo1 (K66)–Syo1 (K35), RpL11 (K76)–RpL11 (K88), Syo1 (K294)–RpL11 (K36)) correlate well with the crystal structure, with the exception of a single inter crosslink that could comprise a false positive identification. Using full-length RpL5 in XL-MS enables us to localize the globular part of RpL5 (residues 45 to 255) that is absent in our crystal structure (Syo1 (K66)—RpL5 (K112); Table 2; Fig. 3a). The RpL5 globular domain localizes in close proximity of the Syo1 N-terminal region. Although the absence of specific crosslinks between RpL5 and RpL11 does not mean that the two proteins are not interacting, a direct interaction can be excluded based on the pull-down experiments (Fig. 2c). Overall, these data permit us to derive a more complete model of the ternary complex.

In the presence of the 5S RNA, the crosslink pattern is the same overall, but shows some specific changes. The number of intralinks at the N-terminal region of Syo1 increased, indicating that the globular domain of RpL5 has moved on binding to 5S RNA and all the previously shielded lysine residues in that area are now free to be crosslinked (Supplementary Fig. 10a,b). The relocation of the RpL5 globular domain is also indicated by the loss of one crosslink (Syo1 (K66)–RpL5 (K112)). Interaction with the 5S RNA also induces changes of the RpL5 N-terminus with respect to Syo1 and a new crosslink is formed (RpL5 (K8)–Syo1 (K642)) suggesting a rearrangement that might facilitate the release of RpL5 from Syo1. Only in the presence of the 5S RNA, intralinks of the RpL5 C-terminus are observed suggesting that this region adopts a defined structure on 5S RNA binding (RpL5 (K276)–RpL5 (K265), RpL5 (K276)–RpL5 (K267)). The RpL5 C-terminal region approaches the Syo1 C-terminus (Syo1 (K642)–RpL5 (K294)), and RpL11 moves with respect to Syo1 in the presence of the 5S RNA, as indicated by the loss of one crosslink (Syo1 (K294)–RpL11 (76)). Taken together, the specific changes observed in the presence of 5S RNA indicate formation of a pre-5S RNP in the presence of Syo1.

## Discussion

Syo1 was identified as a nuclear import adaptor, which allows for the simultaneous import of RpL5 and RpL11 (ref 8). We have previously shown that Syo1 alone passes through the nuclear pore complex by interaction with FG repeats, while the ternary complex requires the importin Kap104 and RanGTP. Kap104 binds to a nuclear localization sequence at the Syo1 N-terminus and addition of RanGTP releases the ternary Syo1 complex, which can bind the 5S RNA^8^. The N-terminus of RpL5 interacts with Syo1 as a linear motif and we provided evidence that RpL11 binding to Syo1 involves the acidic loop. In the current study, we show how Syo1 accommodates its two import cargos on opposite sites of the α-solenoid. Comparison of the three Syo1 structures now available (Syo1, Syo1–RpL5-N, Syo1–RpL5-N–RpL11) visualizes an inherent flexibility of the Syo1 superhelix similar as observed for karyopherins, which accommodate different binding partners by an induced-fit type of mechanism^17^. RpL5-N binding to a groove on the Syo1 surface might shield the FG repeat-binding site or block adjustments necessary for binding. The Syo1-HS at the end of the acidic loop guards the 25S RNA-binding site on RpL11 and can be outcompeted by H84. Intriguingly, many karyopherins contain an acidic loop, which plays an important role in cargo binding and release^17^.

Following a Kap104- and RanGTP-dependent import of the ternary complex into the nucleus, the 5S RNA can bind. The analysis of the 5S RNA interaction with the ternary complex by pull-down experiments, EMSAs and crosslinking MS shows that 5S RNA binding to RpL5 and RpL11 induces rearrangements, indicating the formation of a pre-5S RNP. These data support the idea that Syo1 serves also as an assembly platform for the 5S RNP, and we propose the following working model for 5S RNP biogenesis (Fig. 3c): Syo1 binds its two cargos simultaneously on opposite sides of the α-solenoid, and our data suggest that there is no direct interaction between RpL5 and RpL11 (left panel). The RpL5 N-terminus is accommodated in a groove of Syo1, while RpL11 is recruited via the Syo1-HS. The globular domain of RpL5 is in close proximity of the Syo1 N-terminus, but does not contribute to the interaction as deletion of RpL5-N abolishes dimer formation^8^. The 5S RNA can interact with the ternary complex and, from the experiments with RpL5 deletion variants, the 5S RNA is probably clamped by the RpL5 N- and C-termini in a similar manner as in mature 5S RNP (middle panel). The crosslink data indicate that the globular domain of RpL5 moves with respect to the Syo1 N-terminus and the RpL5 C-terminus approaches the Syo1 C-terminus. RpL11 relocates and binds to the 5S RNA via its basic loop. A pre-5S RNP is built in the presence of Syo1, which may represent an early intermediate in 5S RNP biogenesis. Whether it is directed to the pre-60S ribosomal subunit or whether additional factors (for example, Rpf2 and Rrs1 (ref. 11)) and/or rearrangements are required for maturation is still unknown. A recently described structure of a pre-60S ribosomal subunit contains a mature, assembled 5S RNP, however in a twisted, outward rotated orientation (right panel, bottom)^12^. In this pre-60S, RpL11 is already bound to H84 from the 25S RNA in the same manner as in the mature 60S, highlighting H84 as the primary 5S RNP-docking site^12^. Our data define a new role of Syo1 to specifically shield the H84 interaction site on RpL11. H84 competes with Syo1 for binding to RpL11 (right panel, top), but does not release Syo1 from the pre-5S RNP. For the karyopherins, it was proposed that the energy stored in the distortion of the α-solenoid on cargo binding might enable the disassembly of transport complexes^17^. Although speculative, the energy stored in the distortion of Syo1 on binding of RpL5 might contribute to the release of Syo1. However, further experiments are needed to show at which point Syo1 is released.

Our data suggest that in addition to its role as an import adaptor, Syo1 acts as a molecular chaperone in the ribosome assembly pathway that masks a major binding site for the 60S subunit and prevents unspecific and/or premature RNA interactions. Syo1 copies the H84 interaction with RpL11, and thereby might ensure already in the cytosol that only properly folded RpL11 is imported for 5S RNP biogenesis, and is kept competent for docking to the pre-60S. Quality control of r-proteins before nuclear import and before entering the path of ribosome biogenesis and maturation could be an elegant mechanism to avoid wasteful intermediates.

In essence, our study illustrates the complexity underlying the assembly of an important, but ‘simple' RNP with only three components. It may serve as a model for the understanding of assisted ribonucleoprotein assembly. We expect that similar quality-control mechanisms exist for other r-proteins to avoid nuclear import or recruitment of defective ribosomal components.

## Methods

### Cloning

All recombinant DNA techniques were performed according to established procedures using *Escherichia coli* DH5α for cloning and plasmid propagation. All cloned DNA fragments generated by PCR amplification were verified by sequencing. Plasmids used in this study are listed in Supplementary Table 1.

### Protein preparation and crystallization

The Syo1–RpL5-N–RpL11 complex and the Syo1/RpL5/RpL11 complex were produced as described^8^ and purified by Ni-ion affinity chromatography followed by SEC (S200-26/60, GE Healthcare) in a buffer containing 20 mM HEPES-Na (pH 7.5), 200 mM NaCl, 20 mM KCl, 20 mM MgCl_2_. Crystallization was performed at 291 K by the sitting-drop vapour-diffusion method on mixing equal volumes (0.3 μl) of protein solution (23 mg ml^−1^) and crystallization buffer with a reservoir volume of 100 μl. Crystals appeared within 2 h in 0.1 M sodium acetate, 8% (v/v) PEG 4000.

### Data collection and structure determination

To reduce radiation damage during X-ray data collection, crystals were flash frozen in liquid nitrogen after cryoprotection by transfer into cryosolution containing mother liquor and 20% (v/v) glycerol. Diffraction data were measured using beamline ID23eh2 at the European Synchrotron Radiation Facility (ESRF, Grenoble), at 0.8726 Å under cryogenic conditions (100 K; Oxford Cryosystems Cryostream) using a CCD detector (MAR Research) and processed with the X-ray Detector Software package^23^. The structure was solved by molecular replacement with PHASER^24^ from the CCP4 package^25^, using the structures of Syo1 (PDB: 4GMO^8^) and RpL11 (as part of the yeast ribosome, PDB: 3U5E, chain J^16^) as search models. Model building and refinement was performed with the COOT^26^ and PHENIX suite^27^. Data collection and refinement statistics are summarized in Supplementary Table 1. The model quality was analysed with PROCHECK^28^ and MOLPROBITY^29^. Ramachandran statistics for the final model of Syo1/RpL5-N/RpL11 show 93.23% of residues in most favourable regions, 5.62% in additionally allowed regions and 1.15% in generously allowed or disallowed regions according to PROCHECK^28^. Figures were prepared using the programme PyMOL (Molecular Graphics System, Version 1.5.0.4 Schrödinger, LLC; http://www.pymol.org).

### Preparation of RNA

A synthetic DNA template encoding helix 84 (nucleotides 2,666–2,686, 5′-GGGAGAACAGAAATCTCCC-3′) of yeast 25S RNA, cloned into the pEX-A vector via the EcoRI and HindIII restriction sites, was purchased from Eurofins (MWG Operon). Preparation of the 5S RNA and helix 84 was done as described^8^. After transcription, proteins were removed by phenol/chloroform extraction and the RNA was purified by SEC.

### Electrophoretic mobility shift assays

The RNA-binding activity of the Syo1–RpL5–RpL11 complex was monitored by EMSAs. RNA was folded according to an established protocol^18^. A 0.5-fold molar ratio or an equimolar amount of Syo1–RpL5–RpL11 complex was added to the RNA (5S RNA or H84); then the salt concentration was adjusted to maintain folding buffer conditions and incubated at 25 °C for 10 min. Samples were analysed by Tris-borate agarose electrophoresis in 0.5 × Tris-borate buffer (pH 8.2) for 20 min at 20 mA at room temperature and ethidium bromide staining.

### *In vitro* pull-down assays

Standard methods were used to purify (His)_6_- tagged proteins from *E. coli*. SEC-purified Syo1-FLAG–(His)_6_-RpL5–RpL11 complex was immobilized on anti-FLAG-M2 magnetic beads (Sigma). Equimolar concentrations of folded H84 was added to the immobilized complex on the beads and incubated at 4 °C overnight. After washing with three column volumes of the binding buffer containing 20 mM HEPES-Na (pH 7.5), 150 mM NaCl, 10 mM KCl, 10 mM MgCl_2_, elution was performed by incubation with 0.1 mg ml^−1^ FLAG peptide. Coomassie-stained SDS–polyacrylamide gel electrophoresis revealed the proteins at each step and the RNA was observed by 0.8% agarose electrophoresis and ethidium bromide staining. Similar method was used for the SEC-purified Syo1-FLAG–(His)_6_-RpL5–RpL11–5S RNA complex. As a negative control, buffer was used in place of H84.

Immobilized ion affinity chromatography-purified Syo1–RpL5(His)_6_–GST-RpL11 complex was immobilized on a GSTrap (GE Healthcare). Equimolar concentration of folded H84 was loaded onto the column and incubated at 4 °C overnight. After washing with binding buffer (20 mM HEPES-Na (pH 7.5), 150 mM NaCl, 10 mM KCl, 10 mM MgCl_2_), elution was done with elution buffer (50 mM Tris-HCl, 10 mM reduced glutathione, pH 8.0).

### XL-MS experiments

Syo1–RpL5–RpL11 complex alone and with the 5S RNA were crosslinked with 0.5 mM of H12/D12 isotope labelled disuccimidyl suberate (Creative Molecules) for 30 min at 35 °C under shaking (850 r.p.m.). To generate an XL-MS data set that can be processed as a whole with intrinsically consistent false discovery rate (FDR) estimation, we decided to rely on isotope-coded crosslinkers throughout the entire study. The reaction was quenched by the addition of NH_4_HCO_3_ to 0.1 M for 10 min at 35 °C. Crosslinked proteins were denatured in 4 M urea and 0.1% (w/v) RapiGest Surfactant (Waters), reduced using 10 mM DTT (30 min at 37 °C) and cysteines were carbamidomethylated with 15 mM iodoacetamide (30 min in the dark). Protein digestion was performed first using 1:100 (w/w) LysC (Wako) for 4 h at 37 °C and then finalized with 1:50 (w/w) trypsin (Promega) overnight at 37 °C, after the urea concentration was diluted to 1.5 M. Samples were then acidified with 10% (v/v) trifluoroacetic acid and desalted using MicroSpin columns (Harvard Apparatus) following standard procedures. Crosslinked peptides were enriched using SEC, as described in ref. 20. In brief, desalted peptides were reconstituted with SEC buffer (30% (v/v) ACN in 0.1% (v/v) trifluoroacetic acid) and fractionated using a Superdex Peptide PC 3.2/30 column (GE) on a Ettan LC system (GE) at a flow rate of 50 μl min^−1^. Fractions eluting between 0.9 and 1.3 ml were generally pooled, evaporated to dryness and reconstituted in 20–50 μl 5% (v/v) ACN in 0.1% (v/v) FA according to 215 nm absorbance. Between 2 and 10% of the collected fractions were loaded onto a BEH300 C18 (75 μm × 250 mm, 1.7 μm) nanoAcquity UPLC column mounted on a nanoAcquity UPLC system (Waters) and stepwise eluted with a 3–85% (v/v) ACN in 0.1% (v/v) formic acid gradient connected online to an LTQ-Orbitrap Velos Pro mass spectrometer (Thermo). Data acquisition was performed using a TOP-20 strategy where survey ms scans (*m*/*z* range 375–1,600) were acquired in the orbitrap (*R*=30,000), and up to 20 of the most abundant ions per full scan were fragmented by collision-induced dissociation (normalized collision energy=40, activation *Q*=0.250) and analysed in the LTQ. To focus the acquisition on larger crosslinked peptides, charge states 1, 2 and unknown were rejected. Dynamic exclusion was enabled with repeat count=1, exclusion duration=60 s, list size=500 and mass window ±15 p.p.m. Ion target values were 1,000,000 (or 500 ms maximum fill time) for full scans and 10,000 (or 50 ms maximum fill time) for ms/ms scans. All the samples were analysed in technical duplicates.

Raw files were converted to centroid mzXML using the MassMatrix file conversion too^30^ and analysed using xQuest^21^ based on a FASTA database containing the sequences of the crosslinked proteins. Posterior probabilities and FDRs were calculated using xProphet^22^ and results were filtered using the following parameters: FDR=0.05, min delta score=0.95, MS1 tolerance window ±3 p.p.m.

For crosslink quantification, peaks corresponding to crosslinked peptide ions were manually extracted from raw files using Xcalibur (Thermo). Raw data were queried using the *m*/*z* of the peptide ion identified by xQuest using a mass tolerance of ±20 p.p.m. Peaks were univocally identified based on their retention time, charge state and the presence of both light and heavy peptide ions. Peaks integration was performed using Xcalibur and manually inspected. The integrated area of each peak was then normalized to the average intensity of the sample and used for quantification. The quantification was performed on both technical replicates (repeated injection of the same sample) and the average value was used. We generally observed good reproducibility between technical replicates (average s.d. <12%). In several cases, crosslinked peptide ions were detectable above noise level only in one of the two conditions tested. Such instances were therefore unequivocally assigned only to one condition. When crosslinked peptides were quantified in both the conditions, we used an arbitrary cutoff of twofold change in normalized peak area to determine whether the crosslink was affected by the presence of 5S RNA.

## Author contributions

F.R.C., G.B., K.W., M.B. and I.S. designed the experiments, analysed the data and wrote the manuscript. F.R.C., S.K., A.H. and A.O. performed the experiments. D.K. and E.H. analysed the data and discussed the experiments. All authors read and commented on the manuscript.

## Additional information

**Accession codes:** Protein Data Bank: coordinates and structure factors have been deposited under the accession code: 5AFF.

**How to cite this article:** Calviño, F. R. *et al.* Symportin 1 chaperones 5S RNP assembly during ribosome biogenesis by occupying an essential rRNA-binding site. *Nat. Commun.* 6:6510 doi: 10.1038/ncomms7510 (2015).

## Supplementary Material

Supplementary Information Supplementary Figures 1-10, Supplementary Table 1 and Supplementary References

## Figures and Tables

**Table 1:** Data collection and refinement statistics (molecular replacement).

	**Syo1/RpL5-N/RpL11**
*Data collection*	
Space group	P 2_1_ 2_1_ 2_1_
Cell dimensions	
*a*, *b*, *c* (Å)	60.1, 106.0, 147.2
*α*, *β*, *γ* (°)	90.0, 90.0, 90.0
Resolution (Å)	49.29–3.40 (3.58–3.40)*
*R*_merge_	0.085 (0.581)
*I*/*σI*	15.8 (3.19)
Completeness (%)	99.84 (98.87)
Redundancy	8.1 (8.3)
	
*Refinement*	
Resolution (Å)	49.26–3.40
No. of unique reflections	13,505 (1,308)
*R*_work_/*R*_free_	24.0/29.9
No. of atoms	
Protein	5,620
Ligand/ion	—
Water	—
*B*-factors (Å^2^)	
Protein	141.5
Ligand/ion	—
Water	—
r.m.s. deviations	
Bond lengths (Å)	0.007
Bond angles (°)	1.332

r.m.s., root mean squared.

The following amino-acid residues could not be resolved due to lacking electron density:

Syo1: 24–25; 290–300; 328–384; 536–548; 675–676.

RpL5-N: 1; 24–25; 31–41.

RpL11: 1–14; 50–65; 135–154; 168–173.

^*^Values in parentheses are for highest-resolution shell.

**Table 2:** Quantified crosslinked peptides.

**Crosslinked peptide sequences**	**5S RNA**	
*Intra Syo1*	**−**	**+**
TTAAGAIANIVQDAK(66)CR-LREDK(35)ILPVLK	✓	
HLEEFLPK(636)MR-TLTK(642)GIDK		✓
LLK(147)AQQR-SPDAK(49)SR		✓
ALEHVVPGGAK(294)FNGDAR-LLK(147)AQQR		✓
		
*Intra RpL5*		
AESLK(276)YKK-K(265)SKEEWK		✓
AESLK(276)YK-KSK(267)EEWK		✓
LVK(8)NSAYYSR-QGK(27)TDYYAR	✓	✓
		
*Intra RpL11*		
VLEQLSGQTPVYSK(50)AR-RNEK(65)IAVHVTVR	✓	
GPK(76)AEEILER-VK(88)EYELR	✓	✓
		
*Inter Syo1–RpL5*		
TTAAGAIANIVQDAK(66)CRK-RVLAK(112)LGLDK	✓	
ALEHVVPGGAK(294)FNGDAR-QGK(27)TDYYAR	✓	
TTAAGAIANIVQDAK(66)CR-QGK(27)TDYYAR	✓	
ALEHVVPGGAK(294)FNGDAR-K(257)YVSDAPK		✓
LITQAK(41)NK-LLK(147)AQQR		✓
TLTK(642)GIDKR-VEAK(294)IK		✓
LREDK(35)ILPVLK-LITQAK(41)NK		✓
LREDK(35)ILPVLK-YNAPK(48)YR		✓
LVK(8)NSAYYSR-TLTKGIDK(642)R		✓
QGK(27)TDYYAR-LLK(147)AQQR	✓	✓
ALEHVVPGGAK(294)FNGDAR-LITQAK(41)NK	✓	✓
LREDK(35)ILPVLK-QGK(27)TDYYAR	✓	✓
		
*Inter Syo1–RpL11*		
ALEHVVPGGAK(294)FNGDAR-GPK(76)AEEILER	✓	
AAK(36)VLEQLSGQTPVYSK-ALEHVVPGGAK(294)FNGDAR	✓	✓
