# Supplementary material for: Symportin 1 chaperones 5S RNP assembly during ribosome biogenesis by occupying an essential rRNA-binding site
Source: Nat Commun. 2015 Apr 7;6:6510. doi: 10.1038/ncomms7510 (PMC4396368; doi:10.1038/ncomms7510)
Supplement: Supplementary Information — Supplementary Figures 1-10, Supplementary Table 1 and Supplementary References [file ncomms7510-s1.pdf]

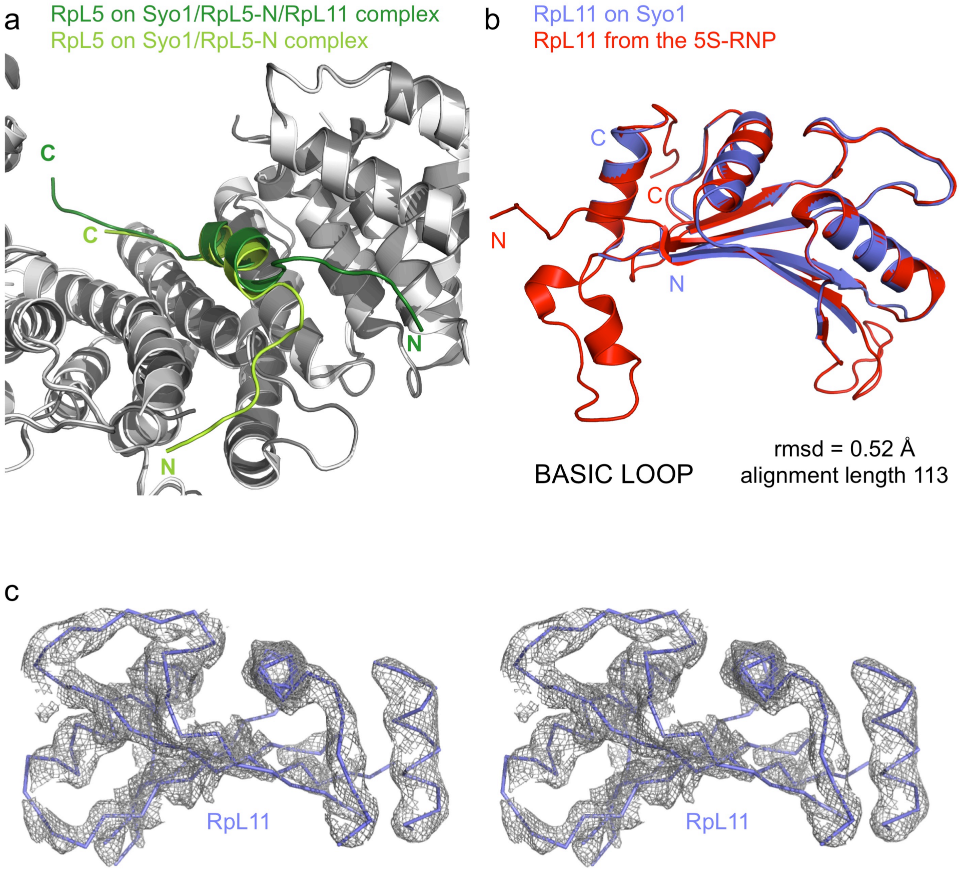

Supplementary Figure 1. Analysis of Syo1 cargos. (a) Superposition of the Syo1–RpL5-N–RpL11 structure (this study; Syo1 is shown in grey), with the previous Syo1–RpL5-N complex (PDB: 4GMN[1]) shows a more extended conformation of the RpL5 N-terminus (green) in the ternary complex. (b) Superposition of RpL11 from 5S RNP (red) as part of the *S. cerevisiae* 80S ribosome (PDB: 3U5E[2]) and RpL11 observed in the Syo1–RpL5-N–RpL11 complex (blue) (this study). RpL11 basic loop involved in 5S RNA binding is disordered in the Syo1 complex. (c) Stereo view of RpL11 (blue) in the Syo1 complex. A 2Fo-Fc electron density map contoured at 1.0 σ .

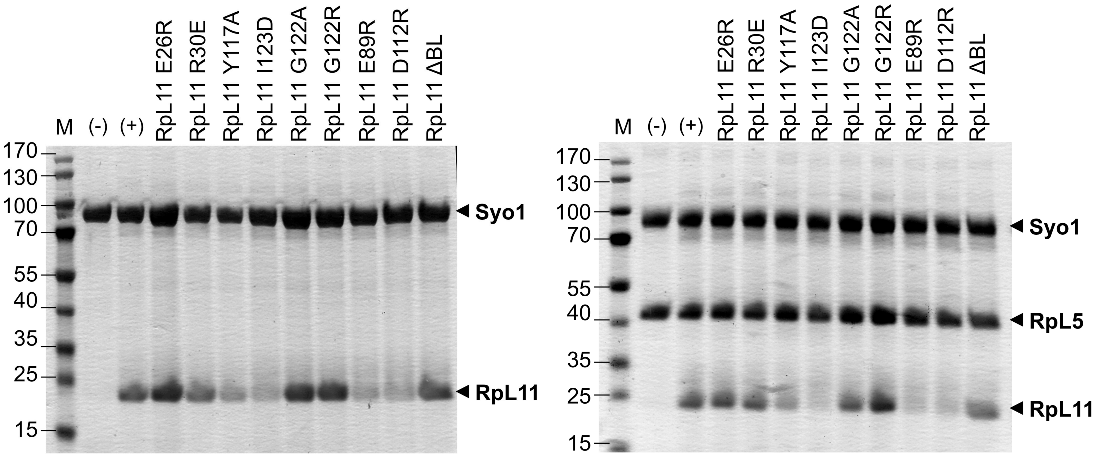

Supplementary Figure 2. Analysis of the RpL11–Syo1 interface by pull-down experiments. RpL11 variants have been tested for Syo1 binding and analyzed by Coomassie-stained SDS-PAGE. The influence of mutations in RpL11 on Syo1 binding is shown in the left panel in the absence of RpL5, and in the right panel in presence of RpL5. In lane (-), Syo1 or the Syo1–RpL5 complex was used as a negative control; lane (+) shows the Syo1–RpL11wt or Syo1–RpL5–RpL11wt complex as a positive control.

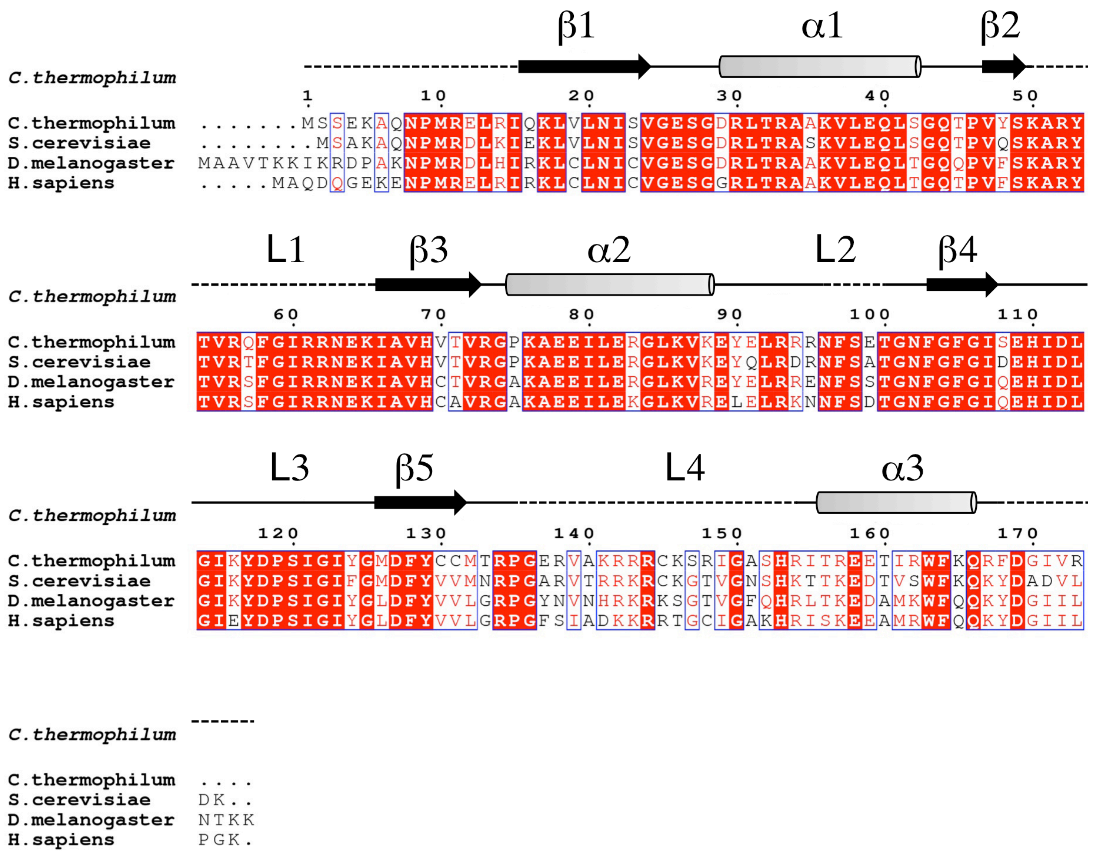

Supplementary Figure 3. Multiple sequence alignments of RpL11. Sequences of representative eukaryotes are shown with structure based secondary structure assignment of *ct*RpL11 (using DSSP[3]). Numbering refers to *ct*RpL11, unstructured regions are shown as dashed lines.

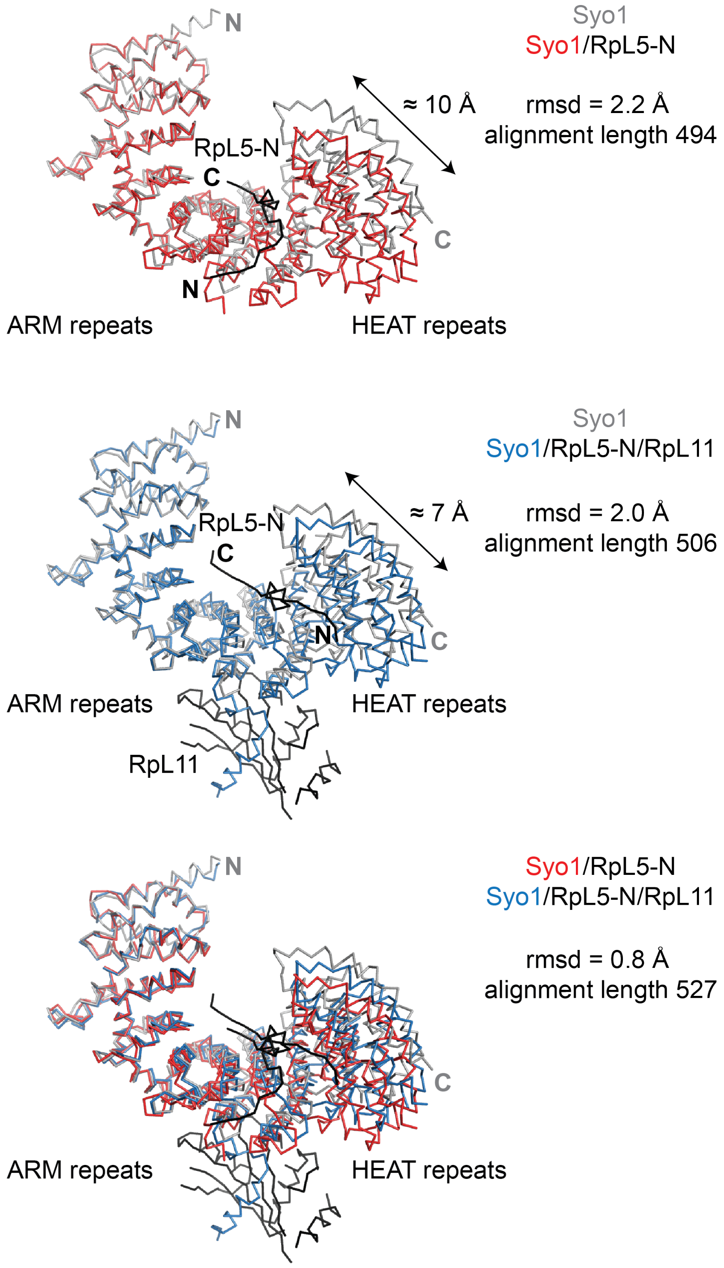

Supplementary Figure 4. Cargo induced changes in Syo1. Three available crystal structures of Syo1 alone (grey) (PDB: 4GMO[1]), Syo1 (red) with bound RpL5-N (black) (PDB: 4GMN[1]) and Syo1 (blue) loaded with both cargos (RpL5-N in black and RpL11 in dark grey) (this study) were superimposed on the ARM domain. While in the Syo1–RpL5-N structure, the HEAT domain of Syo1 is translated by about 10 Å with respect to the ARM domain, this distance is reduced to about 7 Å when both cargos are present. Syo1 alone shows a “closed” conformation, which is “opened” upon accommodation of RpL5-N. In the presence of RpL5-N and RpL11, Syo1 again adjusts.

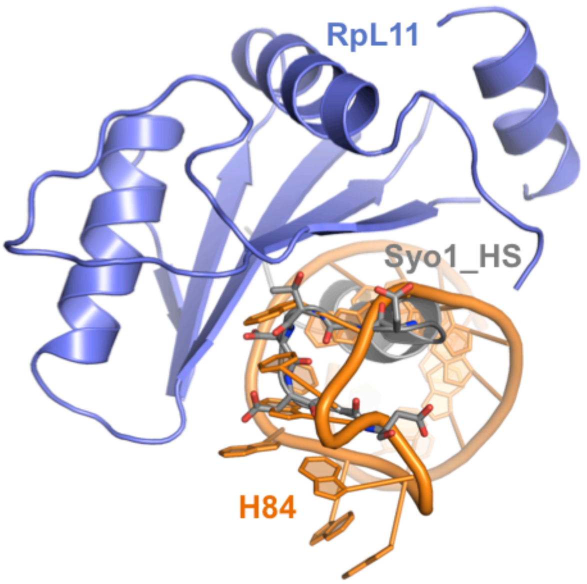

Supplementary Figure 5. Superposition of RpL11 bound to Syo1 or helix 84. The concave surface of RpL11 (purple) is occupied in the Syo1–RpL5-N–RpL11 complex by the Syo1-HS (grey), but in the context of the large ribosomal subunit (60S), it is interacting with H84 (orange) of the 25S RNA[2]. The Syo1-HS (residues 389-399) shares molecular details with its RNA counterpart.

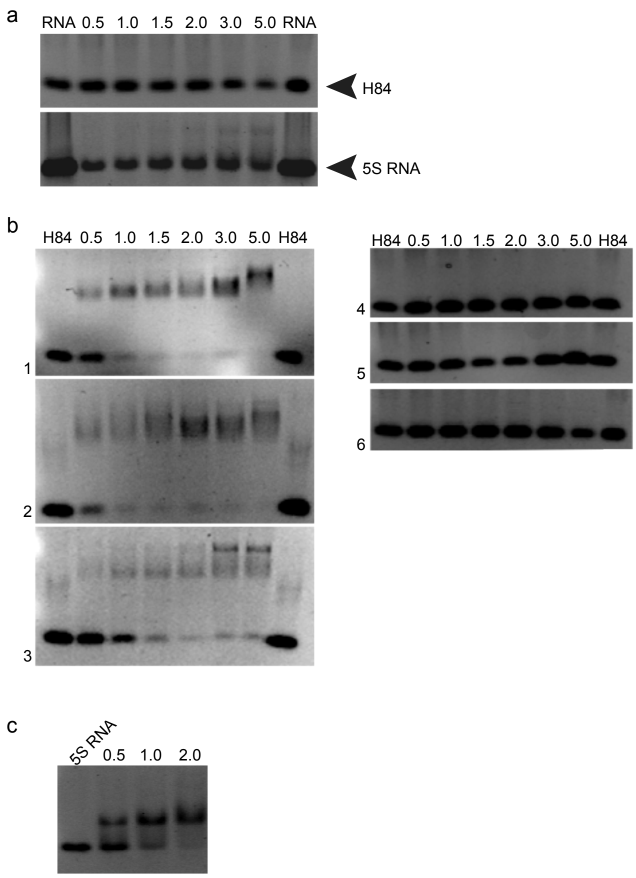

Supplementary Figure 6. EMSA titration experiments. (a) Syo1 does not bind RNA. EMSAs performed with increasing amounts of Syo1 show no interaction with H84 or 5S RNA. 'RNA' lanes are free of protein to provide a base line for the shift. The protein/RNA ratio is given at the top. Same for (b) and (c). (b) Binding of H84 to the truncation variants of RpL5 and RpL11 (as in Fig. 2b) was tested. The complete titrations are shown for Syo1–RpL5–RpL11 (panel 1), Syo1–RpL5–RpL11ΔBL (panel 2), Syo1–RpL5ΔC–RpL11ΔBL (panel 3), Syo1–RpL5-N–RpL11 (panel 4), Syo1–RpL11 (panel 5), and Syo1–RpL11ΔBL complexes (panel 6). (c) Analysis of 5S RNA binding to the Syo1–RpL5ΔC complex. The EMSAs confirm that RpL5 N-terminus and globular domain are sufficient for 5S RNA binding.

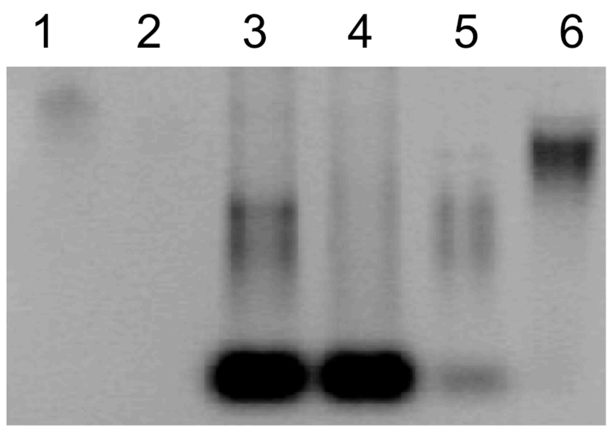

Supplementary Figure 7. Analysis of samples from the GST-pull down experiment (see Fig. 2c) **by an ethidium bromide stained agarose gel.** Lane 1, Syo1–RpL5–GST-RpL11 complex; lane 2, wash with binding buffer; lane 3, flow through after loading H84 onto the column; lane 4, wash with binding buffer; lane 5, additional wash with binding buffer; lane 6, elution. H84 elutes with RpL11 - indicating that it competes with Syo1 for binding to RpL11.

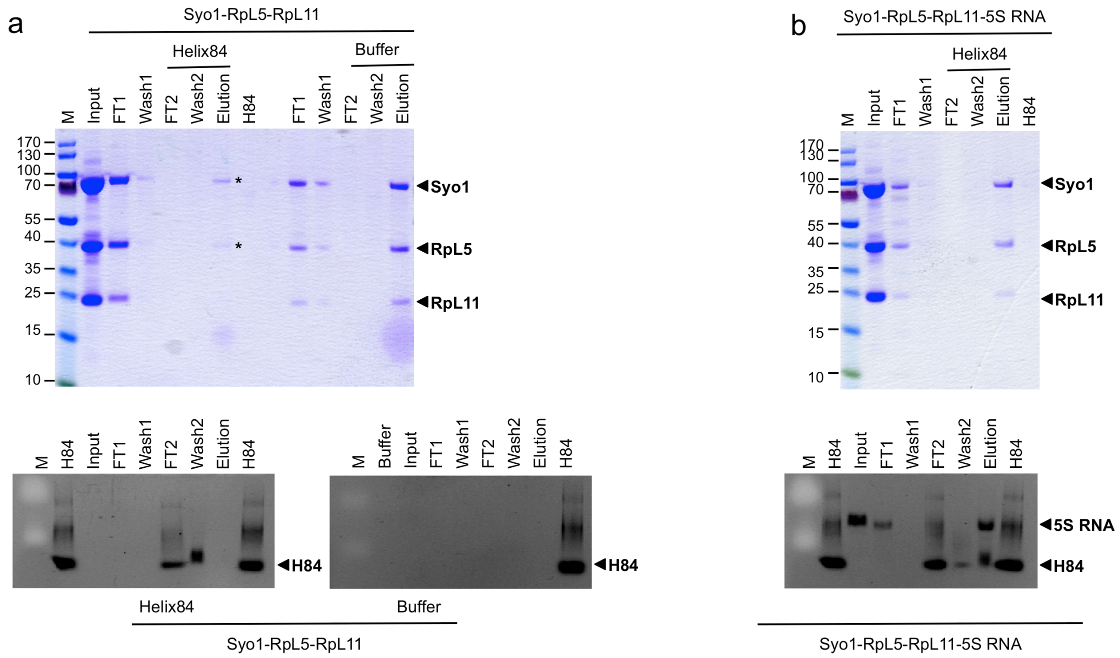

Supplementary Figure 8. Analysis of helix 84 interaction with RpL11 in an *in vitro* pull-down assay using FLAG-tagged Syo1. Upper panels show the Coomassie stained SDS PAGE, lower panels the corresponding ethidium bromide stained agarose gels. H84 was added to the Syo1–RpL5–RpL11 complex (a) in the absence of the 5S RNA. H84 competes with Syo1 for interaction with RpL11. Release of RpL11 from the ternary complex is indicated by its absence from the elution fraction (with H84, upper panel), and confirmed by the absence of H84 in the elution fraction (lower panel). M, molecular weight standard; Input, SEC purified complex; FT1, flow through fraction after loading the complex; W1, wash fraction with binding buffer; FT2, flow through fraction after loading H84; W2, wash fraction with binding buffer; Elution, elution with FLAG peptide. Black arrowheads indicate positions of Syo1, RpL5 and RpL11. As a negative control, binding buffer was used instead of H84. RpL11 released was not observed. (b) in the presence of the 5S RNA. Pull-down experiments were repeated with a stable SEC purified Syo1-FLAG–RpL5–RpL11–5S RNA complex. H84 is not able to release RpL11 from the quaternary complex and recruitment of H84 is indicated by its presence in the elution fraction (lower panel).

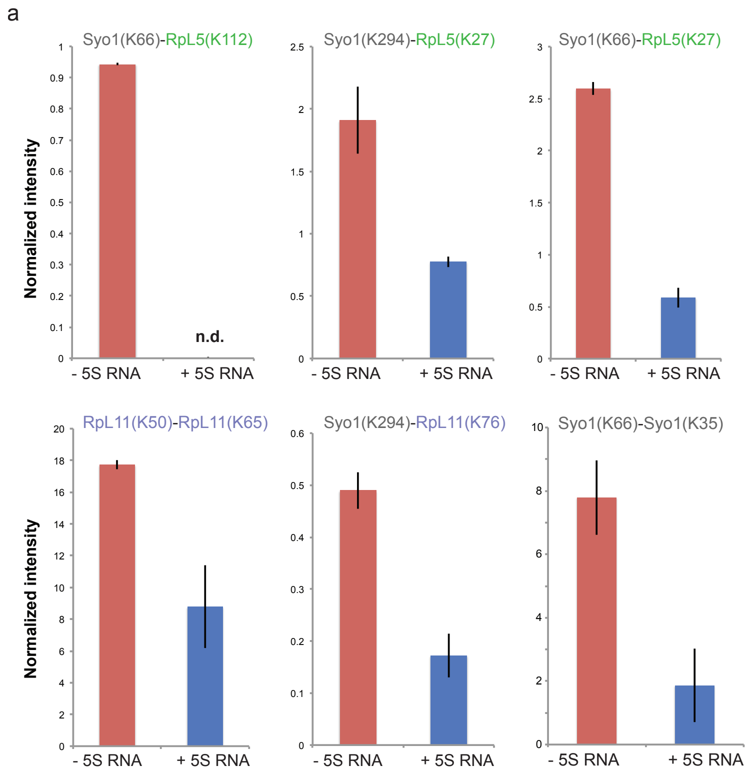

Supplementary Figure 9. Histograms of cross-link quantification. (a) Cross-links with higher normalized intensity in the sample without 5S RNA.

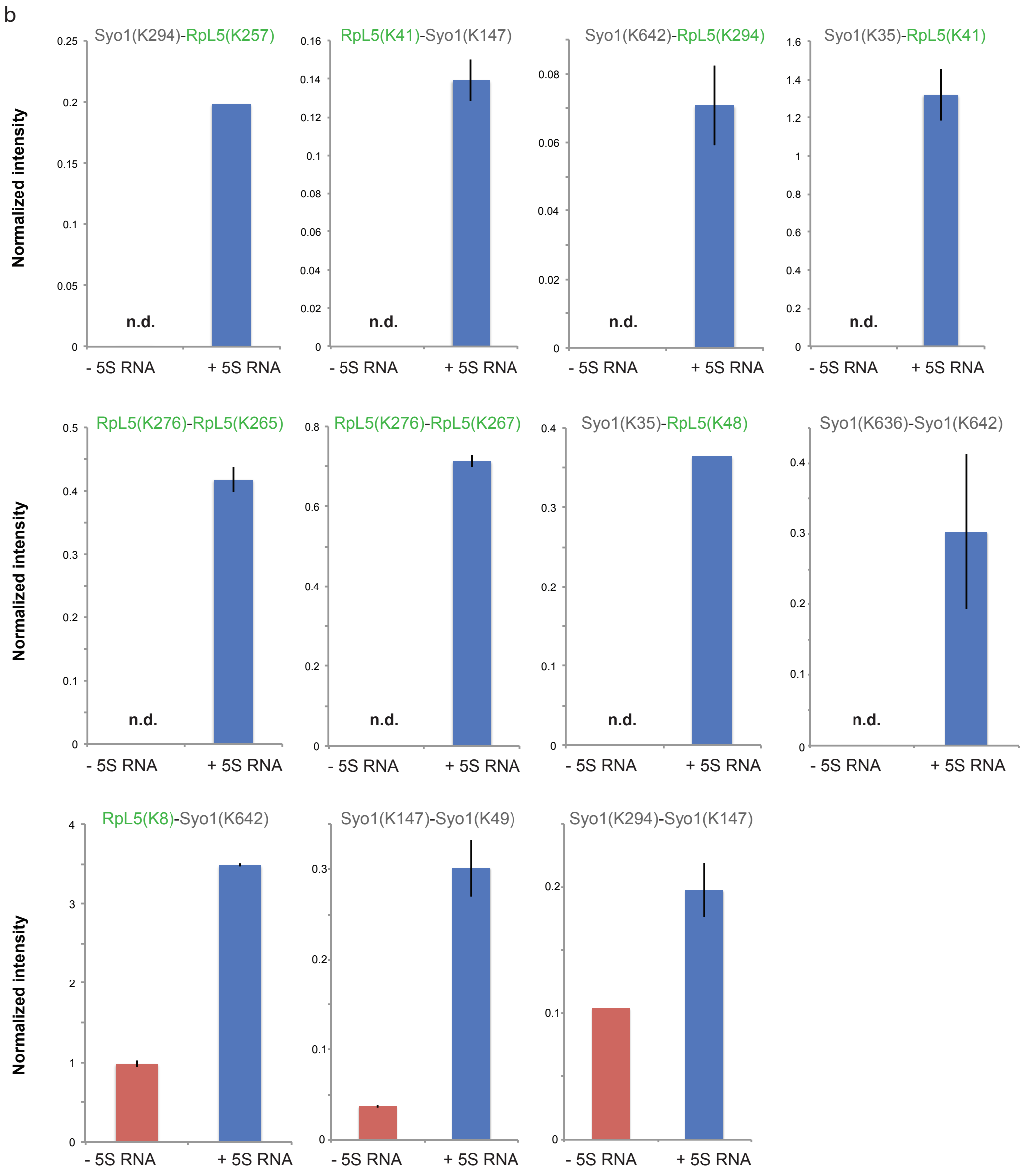

Supplementary Figure 9. Histograms of cross-link quantification. (b) Cross-links with higher normalized intensity in the sample with 5S RNA.

c

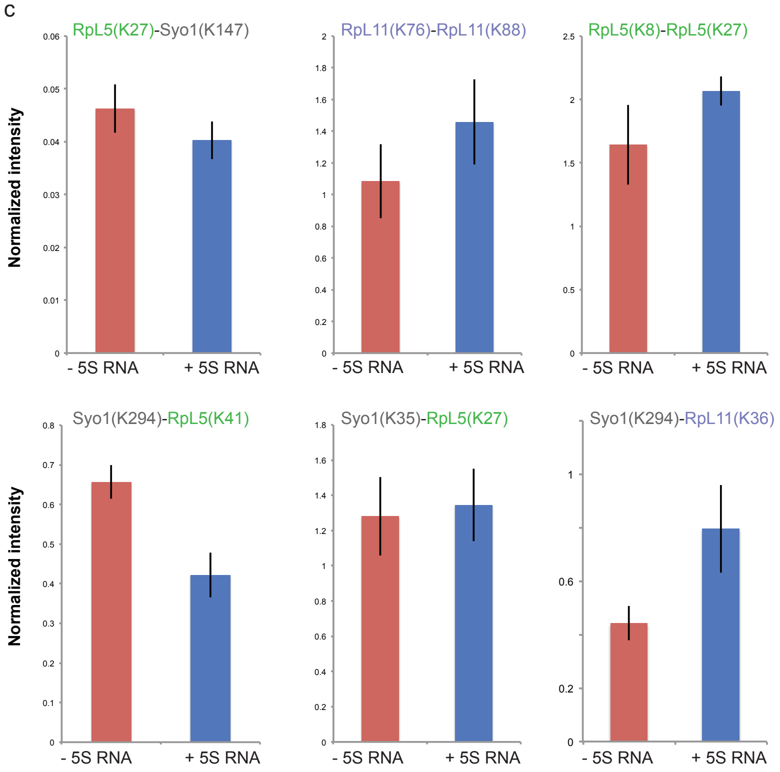

Supplementary Figure 9. Histograms of cross-link quantification. (c) Cross-links with similar normalized intensity in both samples.

Cross-linked peptides were quantified by manual peak extraction as described in Supplementary Methods. Peptide intensities were normalized to the average intensity of the sample and they are displayed as average values ± standard deviation across two technical replicates. Some peptides could not be detected above noise level in one of the two conditions tested and they are indicated as “n.d.”. If the normalized intensity of a cross-link in one sample is, at least, double the value of the other sample, this cross-link will be considered as unique to this sample. Red bars indicate sample without 5S RNA, while blue ones represent sample with 5S RNA.

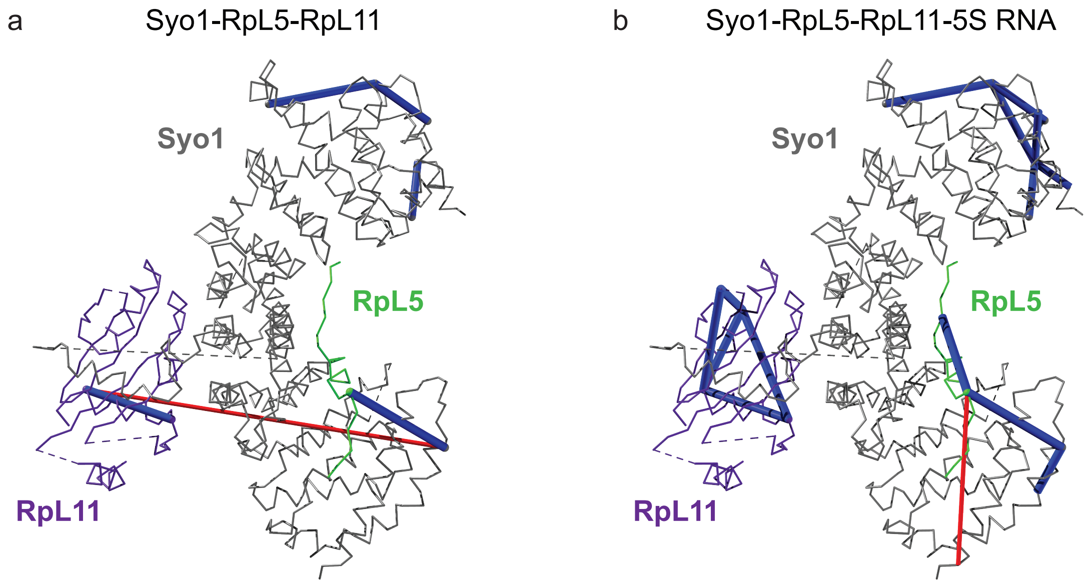

Supplementary Figure 10. Schematic representation in our X-ray structure of the different cross-links obtained (a) in the absence and (b) in presence of the 5S RNA. Blue lines represent cross-links within a 30Å Cα-Cα distance in the structure. Red lines show cross-links that cannot be reconciled with this particular structural arrangement, as they correspond to distances greater than 30Å; in the absence of 5S RNA, RpL11 and Syo1 show a deviation (red line in a) that likely represents a false positive identification. In the presence of 5S RNA, there is also a violated intra-protein cross-link at the N-terminus of RpL5. It appears that for the 5S RNA binding, the N-terminus of RpL5 needs to move out of the groove, getting closer to C-terminus of Syo1 (red line in b).

Supplementary Table 1.

Table 1. *E. coli* expression plasmids used in this study*.

Name	Relevant information	Source
pET15b/SYO1-FLAG-$(His)_6$	amp^r, T7 promoter/lac operator	Kressler et al.[1]
pETDuet-1/RPL5(1-41)-$(His)_6$-SYO1(24-676)	amp^r, T7 promoter/lac operator	This study
pET24d/RPL11	kan^r, T7 promoter/lac operator	Kressler et al.[1]
pETDuet-1/RPL5-SYO1-FLAG	amp^r, T7 promoter/lac operator	Kressler et al.[1]
pETGST-1a/$(His)_6$-GST-TEV-RPL11	kan^r, T7 promoter/lac operator	This study
pETDuet-1/RPL5-$(His)_6$-SYO1-FLAG	amp^r, T7 promoter/lac operator	Kressler et al.[1]

*For simplicity, mutants are not listed since they were cloned into the listed plasmids.

Supplementary References

1. Kressler, D. et al. Synchronizing nuclear import of ribosomal proteins with ribosome assembly. *Science* **338**, 666-71 (2012).
2. Ben-Shem, A. et al. The structure of the eukaryotic ribosome at 3.0 A resolution. *Science* **334**, 1524-9 (2011).
3. Kabsch, W. & Sander, C. Dictionary of protein secondary structure: pattern recognition of hydrogen-bonded and geometrical features. *Biopolymers* **22**, 2577-637 (1983).
